# Advanced Mass Spectrometry-Based Biomarker Identification for Metabolomics of Diabetes Mellitus and Its Complications

**DOI:** 10.3390/molecules29112530

**Published:** 2024-05-27

**Authors:** Feixue Zhang, Shan Shan, Chenlu Fu, Shuang Guo, Chao Liu, Shuanglong Wang

**Affiliations:** 1 Hubei Key Laboratory of Diabetes and Angiopathy, Medicine Research Institute, Medical College, Hubei University of Science and Technology, Xianning 437100, China; feixuezhang@hbust.edu.cn (F.Z.); fuchenlu2022@163.com (C.F.); guoshuang@hbust.edu.cn (S.G.); 2 College of Life Science, National R&D Center for Freshwater Fish Processing, Jiangxi Normal University, Nanchang 330022, China; 005274@jxnu.edu.cn; 3 School of Pharmacy, Medical College, Hubei University of Science and Technology, Xianning 437100, China; 4 Jiangxi Key Laboratory for Mass Spectrometry and Instrumentation, East China University of Technology, Nanchang 330013, China

**Keywords:** mass spectrometry, diabetes mellitus, diabetes complications, metabolomics, LC-MS, GC-MS

## Abstract

Over the years, there has been notable progress in understanding the pathogenesis and treatment modalities of diabetes and its complications, including the application of metabolomics in the study of diabetes, capturing attention from researchers worldwide. Advanced mass spectrometry, including gas chromatography–tandem mass spectrometry (GC-MS/MS), liquid chromatography–tandem mass spectrometry (LC-MS/MS), and ultra-performance liquid chromatography coupled to electrospray ionization quadrupole time-of-flight mass spectrometry (UPLC-ESI-Q-TOF-MS), etc., has significantly broadened the spectrum of detectable metabolites, even at lower concentrations. Advanced mass spectrometry has emerged as a powerful tool in diabetes research, particularly in the context of metabolomics. By leveraging the precision and sensitivity of advanced mass spectrometry techniques, researchers have unlocked a wealth of information within the metabolome. This technology has enabled the identification and quantification of potential biomarkers associated with diabetes and its complications, providing new ideas and methods for clinical diagnostics and metabolic studies. Moreover, it offers a less invasive, or even non-invasive, means of tracking disease progression, evaluating treatment efficacy, and understanding the underlying metabolic alterations in diabetes. This paper summarizes advanced mass spectrometry for the application of metabolomics in diabetes mellitus, gestational diabetes mellitus, diabetic peripheral neuropathy, diabetic retinopathy, diabetic nephropathy, diabetic encephalopathy, diabetic cardiomyopathy, and diabetic foot ulcers and organizes some of the potential biomarkers of the different complications with the aim of providing ideas and methods for subsequent in-depth metabolic research and searching for new ways of treating the disease.

## 1. Introduction

**Figure 1:**
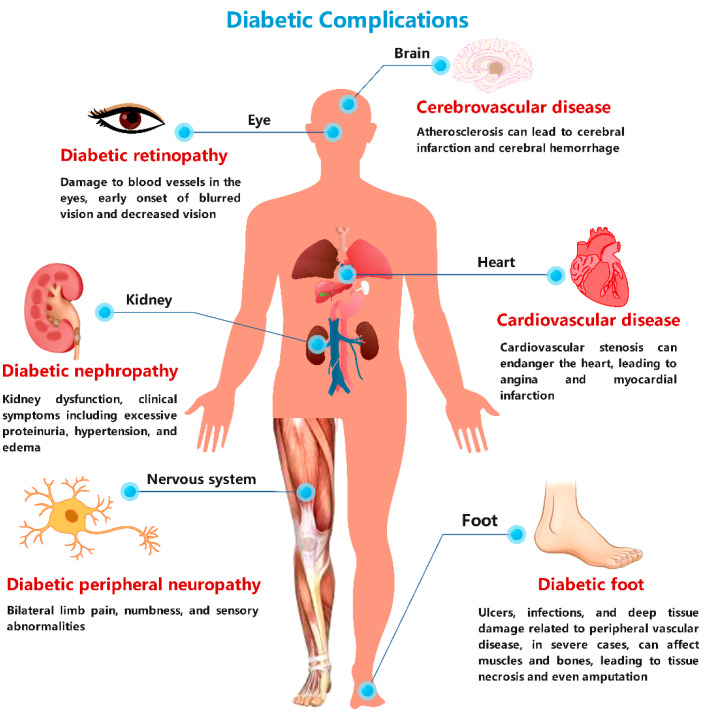
Diabetes complications.

Diabetes mellitus (DM) is a persistent metabolic disorder caused by intricate interplays between genetic factors and environmental influences. Its high morbidity, mortality, and complications are primarily attributed to prolonged hyperglycemia and insulin resistance, disrupting normal glucose and lipid metabolism. With socioeconomic development and lifestyle changes, diabetes has emerged as a significant chronic ailment posing a grave threat to human health. Currently, the incidence of diabetes mellitus is rising year by year. The statistical results of the Non-communicable Disease Risk Factor Collaboration in cooperation with the World Health Organization show that in 1980 about 108 million people worldwide suffered from diabetes [1] and that in 2021 the number of diabetic patients reached 537 million; it is expected that by 2050 the number of people suffering from diabetes will be over 1.31 billion [2]. The prevalence of impaired glucose tolerance reached 374 million in 2019, or approximately 7.5% of the global population, with prediabetes incidence set to rise to 548 million, or about 8.6 percent of the global adult population, by 2045. Statistics for 2019 show that the total global healthcare costs for diabetes were as high as USD 760 billion [3], and in 2021 global health expenditure reached USD 966 billion and is expected to exceed USD 1054 billion by 2045 [2]. The occurrence of diabetes is on the rise globally, affecting both developed and developing nations. Prediabetes comprises impaired glucose tolerance and impaired fasting glucose, with the former being three times more prevalent than the latter. Prolonged hyperglycemia and insulin resistance will cause abnormalities in glucose and lipid metabolism, which will lead to a variety of complications (Figure 1), including diabetic nephropathy, cardiovascular disease, cerebrovascular disease, retinopathy, diabetic foot, and neuropathy. For example, about one-third of diabetic patients will experience retinopathy [4], the probability of developing cardiovascular disease is two to three times greater than normal [5], the probability of developing diabetic foot is five times higher than normal [6], and the probability of developing diabetic chronic kidney disease is two times higher than normal [7]. As diabetes continues to progress, so does the likelihood of complications. For example, diabetic patients are three times more likely to experience infective endocarditis after 15 years of diabetes than when they first developed diabetes [8]. In particular, cardiovascular and renal complications from diabetes have become the most common cause of death in people with diabetes. Even more frighteningly, a person’s limb is amputated due to diabetes every 3 s worldwide [9]. Type 2 diabetes mellitus (T2D or T2DM) constitutes around 90% of diabetic cases, and its pathogenesis is influenced by complex genetic and environmental factors that are not completely understood yet. Many researchers have conducted extensive studies on type 2 diabetes [10,11,12,13] and demonstrated the close relationship between diabetes mellitus and insulin levels. Insulin promotes the synthesis of proteins, fats, and glycogen, leading to alterations in the body’s small-molecule metabolites, such as sugars, amino acids, carnitine, fatty acids, and nucleotides. Metabolomics systematically investigates changes in metabolite composition, contents, and levels in the organism, providing novel insights into the pathogenesis of T2DM. Current research in metabolomics has revealed significant alterations in metabolite profiles associated with different diabetic states, including impaired glucose tolerance, impaired fasting glucose [13], and T2DM. While much focus has been on the diabetic stage, there is a need for more studies on prediabetes mellitus. In light of this, a basic understanding of metabolomics and its analytical techniques is essential.

**Figure 2:**
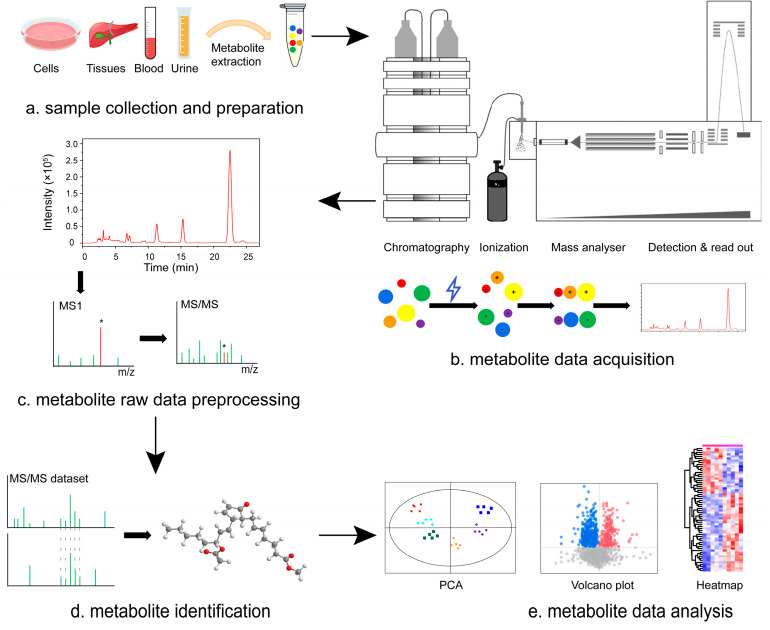
The basic process of metabolomics research. Different colors generally refer to different components of complex substances; * in part c represent a certain target component; black arrows point to the next step; PCA, principal component analysis.

Metabolomics constitutes a vital component of systems biology, specifically concentrates on endogenous metabolites with a molecular weight of less than 1.5 kDa [14], and explores the diverse metabolic responses of biological systems to external environmental stimuli, genomic mutations or modifications, pathological triggers, and physiological changes [15,16,17]. Advancements in research have enabled the application of metabolomics for high-throughput analysis of small molecules in biological specimens. This analytical approach facilitates the prediction of metabolite levels within the body and timely diagnosis of physiological states. Consequently, metabolomics assumes a pivotal role in the prevention, diagnosis, and treatment of prediabetes and diabetes-related conditions [18,19]. The results of previous studies showed that increase in branched-chain amino acids (BCAAs) was negatively correlated with insulin sensitivity and insulin metabolic clearance and positively correlated with fasting insulin through untargeted metabolomics detection of BCAAs [20]. Thus, it is proved that BCAAs is related to insulin resistance and type 2 diabetes [21,22]. Similarly, altered phospholipid metabolites and distortions of lipoprotein metabolism have been demonstrated to exhibit associations with insulin resistance and T2DM [23,24]. Furthermore, metabolomic analyses revealed elevated levels of certain sugar metabolites and sugar derivatives in prediabetic individuals compared to their non-diabetic counterparts [25]. Moreover, multiple amino acids, including aromatic amino acids, glycine, glutamine, and glutamate, have been shown to be associated with prediabetic symptoms and an increased risk of developing type 2 diabetes [26,27]. Expanding the current understanding of the physiology and pathology of type 2 diabetes and identifying new potential biomarkers may help to promote the detection of diabetes [13]. However, the wide variety of metabolites in organisms, coupled with their complex structures and significant differences in content, pose numerous challenges for existing analytical methods. These challenges include limitations in metabolite monitoring coverage, mass spectrometry information coverage, as well as issues related to qualitative and quantitative accuracy [28,29,30]. To address these challenges, it is imperative to drive technological innovations and methodological breakthroughs in all facets of metabolomics technology. In this context, it is imperative to first acquaint oneself with the analytical techniques pertinent to metabolomics. The basic process of metabolomics research is illustrated in Figure 2, which includes biological sample collection, sample preprocessing, metabolite detection, data processing, and biological analysis. Currently, the core detection technologies for the metabolome of biological organisms are nuclear magnetic resonance (NMR) and mass spectrometry (MS) [31].

A typical metabolomics experiment can be delineated into several key steps. (a) Sample collection and preparation. Samples are collected from selected individuals, and this should be carried out under stringent clinical conditions to ensure the integrity and representativeness of the samples. Then, the samples are physically or chemically treated to extract metabolites, which may involve protein precipitation, centrifugation, filtration, and other purification steps to make the sample suitable for downstream analysis. (b) Metabolite data acquisition. Untargeted metabolomics analysis: In this phase, LC-MS/MS is employed for a comprehensive analysis of a wide array of metabolites present in the samples. Targeted metabolomics analysis: This utilizes the same LC-MS/MS technology but focuses specifically on certain metabolites. Targeted metabolomics and untargeted metabolomics have different research objectives. Targeted metabolomics focuses on multiplex analysis of known metabolites and absolute qualitative and quantitative analysis of the substance to be tested using standards. Untargeted metabolomics analyzes all metabolites in an organism or system in a high-throughput manner to find and screen key metabolites for subsequent analysis. In short, the research object of targeted metabolomics is determined before the experiment, while the research object of untargeted metabolomics is not determined before the experiment. Therefore, in practical applications, it is necessary to choose according to the specific experimental purpose. If you want to obtain as much information about metabolites as possible, you can choose the method of untargeted metabolomics; if you want to obtain absolute qualitative and quantitative data on specific metabolites, it is suitable to choose the method of targeted metabolomics. (c) Metabolite raw data preprocessing. The raw data generated by LC-MS/MS are processed using appropriate software and algorithms to identify and quantify the metabolites in the samples. (d) Metabolite identification. The data obtained are analyzed using statistical and bioinformatics methods, aiming to identify significant changes in metabolites. (e) Metabolite data analysis. To ensure accuracy and reproducibility, the findings typically undergo validation across different sample sets; this may include multivariate analysis, trend analysis, and comparisons with healthy control groups. (The images in part e in the above figure were adapted from the pre-experimental data of our research group and do not have practical application value.)

Nuclear magnetic resonance (NMR) spectroscopy is a versatile analytical technique that utilizes static and radiofrequency magnetic fields to generate images based on the differences in energy attenuation of specific nuclei. It holds a crucial role in metabolomics research due to its distinctive advantages, including straightforward sample handling, non-destructive properties, a rich array of analytical tools, qualitative and quantitative capabilities, unbiasedness, and rapid analysis [32]. Del et al. utilized 1H-NMR to discriminate between serum metabolic profiles in individuals with T2DM and in individuals with T2DM complications; they identified eight markers in T2DM patients, including five metabolites, namely, isoleucine, leucine, lysine, tyrosine, and valine, which could potentially serve as biomarkers for T2DM complications [33]. In a separate study, Palomino et al. employed 1H-NMR to investigate the metabolic profiles of erythrocytes in T2DM patients [34]. They discovered differential metabolites, including amino acids like glutathione, 2,3-bisphosphoglyceric acid, and inosinic acid, in comparison with those in the healthy population. In recent years, the development of nuclear magnetic resonance technology has greatly improved our understanding of diabetic retinopathy (DR) [35], diabetic nephropathy (DN) [36], and cerebrovascular neuropathy [37]. It has become an important tool for monitoring metabolic changes and predicting disease progression. However, due to the low sensitivity, signal overlap in complex substrates, and the high cost of instrument use and maintenance, the detection of millimole metabolites is challenging, which limits the application of NMR in large-scale metabolomics research [38,39]. In recent years, LC-MS-based and GC-MS-based metabolomics have been widely used and developed for the discovery of metabolite markers of disease, which has created opportunities for the discovery of new T2D biomarkers [40]. Such progress could enhance our understanding of the metabolic mechanisms underlying the onset and progression of T2DM and assist in the identification of early potential metabolic markers for the condition [41]. The primary objective of this review is to summarize the detection technologies used in diabetes metabolomics, analytical techniques in metabolomics, and the application of MS-based metabolomics in diabetes and its complications. Its purpose is to provide readers with an entry point into the field of metabolomics related to diabetes and its complications for the first time.

## 2. MS Technology for Biomarker Identification in Metabolomics of Diabetes Mellitus and Its Complications

The MS detection technique ionizes sample components within an ion source, generating ions with different mass-to-charge ratios (*m*/*z*). These varying *m*/*z* ions are separated in a mass spectrometer using magnetic or electric fields and then sequentially measured in a detector. Mass spectra provide detailed *m*/*z* and mass information after signal amplification and processing. Compared to NMR, MS offers high sensitivity (pM-fM), analysis of a wide range of substances in a single determination, and high specificity. However, MS cannot detect metabolite molecules that are not easily ionized, and the sample pretreatment process can be cumbersome [42]. Due to the intricate composition of metabolites in biological systems, encompassing numerous species and significant variations in physicochemical properties, it is crucial to carefully select a suitable MS analysis platform in metabolomics studies, depending on the experimental purpose or the type of metabolite under investigation. Among common MS ion sources, as listed in Table 1, electron ionization (EI), chemical ionization (CI), electrospray ionization (ESI), atmospheric pressure chemical ionization (APCI), and matrix-assisted laser desorption ionization (MALDI) are included [43]. The EI source uses a high-energy electron beam to collide with compounds, producing molecular ions. These ions may undergo further fragmentation due to the breakage or rearrangement of internal chemical bonds upon collision with electrons or helium gas. EI is particularly suitable for analyzing thermally stable and volatile substances and is often used in conjunction with gas chromatography (GC). The CI source, a soft ion source, introduces reactive gas in addition to the EI process. The high-energy electron beam preferentially ionizes the reactive gas, which then ionizes the compound through reactions between the gas ions and the compound. ESI, functioning under atmospheric pressure, generates charged droplets containing compounds. ESI is ideal for volatile and thermally unstable substances and is commonly paired with liquid chromatography (LC) or GC. APCI ionizes oxygen or nitrogen molecules through continuous corona discharge inside the source. The charged ions transfer their charge to solvent molecules, which then ionize the compound molecules, making them suitable for analyzing volatile and heat-stable substances. MALDI involves dispersing compounds in a matrix to form a cocrystal. When a laser beam hits the crystal, the matrix molecules absorb photon energy, causing the mixture to transition from a solid to a gaseous state, facilitating charge transfer and compound ionization. MALDI is optimal for analyzing large molecules.

Mass spectrometry is classified into high-resolution mass spectrometry (HRMS) and low-resolution mass spectrometry (LRMS) based on the resolution of the compound’s *m*/*z*. HRMS has a resolution greater than 10,000 and typically includes time-of-flight mass spectrometry (TOF-MS), Fourier transform ion cyclotron resonance mass spectrometry (FTICR-MS), electrostatic Orbitrap mass spectrometry, and so on. HRMS offers significant advantages in metabolite characterization, allowing precise acquisition of metabolite primary *m*/*z* and isotope peaks, as well as high-quality MS/MS. The classic technique, mass spectrometry coupled with chromatography, can effectively reduce matrix effects and achieve isomer separation to a certain extent, which makes it popular in the analysis of complex samples. Commonly used chromatography–mass spectrometry platforms include GC-MS and LC-MS.

In the realm of metabolomics, LC-MS stands as a pivotal analytical technique [44]. LC-MS unites the separation capabilities of LC with the mass analysis power of MS. This synergy allows for the precise identification and quantification of a vast array of metabolites in complex biological samples. The strength of LC-MS lies in its versatility and sensitivity. Different LC modalities, such as reverse-phase hydrophilic interaction liquid chromatography (HILIC) and ion-exchange chromatography, can be employed to separate metabolites based on their distinct properties, such as polarity, size, or ionic charge. Once separated, the metabolites are ionized (commonly using ESI) and then analyzed by MS [45]. This results in highly specific mass-to-charge (*m*/*z*) ratios that facilitate the accurate identification of metabolites. LC-MS is particularly adept at analyzing non-volatile, thermally labile, and large organic molecules, making it an indispensable tool for profiling complex biological matrices. Its application extends from biomarker discovery to pathway analysis, offering profound insights into the metabolic alterations associated with diseases, environmental exposures, and genetic modifications.

GC-MS is another cornerstone technique in metabolomics, renowned for its efficacy in analyzing volatile and semi-volatile compounds [46]. GC-MS combines the separation power of GC with the detection capabilities of mass spectrometry. In GC-MS, metabolites are first derivatized to enhance their volatility and thermal stability. The derivatized metabolites are then vaporized and carried through a capillary column by an inert gas (usually helium). The separation in GC is primarily based on the compound’s boiling point and affinity to the column material [47]. Following separation, the compounds are ionized (typically using EI) and then detected by MS, providing distinct mass spectra for compound identification. GC-MS is highly sensitive and capable of quantifying trace-level metabolites. It excels in the analysis of small organic molecules like fatty acids, amino acids, and organic acids. Its application ranges from environmental metabolomics to clinical diagnostics, playing a critical role in uncovering metabolic pathways and disease biomarkers.

MS and tandem mass spectrometry (MS/MS) techniques offer significant advantages in metabolomics research, which include the following [48]: (1) high sensitivity and specificity: MS and MS/MS technologies are capable of detecting metabolites at extremely low concentrations, which is crucial for identifying and quantifying rare or low-abundance metabolites within complex biological samples. The specificity is further enhanced by MS/MS through the selection and fragmentation of precursor ions, allowing for accurate identification of target molecules; (2) broad dynamic range: these technologies can detect metabolites across a wide concentration range, enabling the analysis of both high- and low-abundance metabolites, thus providing comprehensive metabolomic coverage; (3) structural elucidation capabilities: particularly with MS/MS, the fragmentation of molecules yields vital information regarding molecular structures. This is invaluable for the identification and structural characterization of unknown metabolites, facilitating the discovery of new metabolic pathways and biomarkers; (4) accurate quantitative analysis: MS techniques, especially when used in conjunction with internal standards, allow for precise quantification of metabolites. This is essential for understanding metabolic changes and disease mechanisms; (5) high-throughput analysis capability: suitable for high-throughput analysis, MS technologies can process and analyze large numbers of samples in a short period of time and can directly analyze complex biological substrates, such as blood, urine, and tissue extracts, which are critical for large-scale metabolomics studies and biomarker screening; (6) versatility and flexibility: MS and MS/MS are applicable to a wide range of analytical targets, including small-molecule metabolites, proteins, and lipids, serving diverse research fields and objectives. The most comprehensive coverage of metabolomic information can be achieved by integrating multiple analysis platforms. With the ongoing advancement of metabolomics technology and in-depth research, the significance of metabolic markers in clinical diagnosis, disease prognosis, and pathological research has gained prominence. In metabolomics research, various analysis strategies can be chosen based on the experimental objectives and specific analytical needs, including untargeted and targeted metabolomics analysis strategies [49]. Different analysis strategies possess distinct focuses and characteristics. Choosing the appropriate analysis strategy according to the experimental goals is of paramount importance in achieving meaningful and reliable results.

In untargeted metabolomics analysis, there is no prerequisite knowledge of specific metabolites in a sample. The strategy aims to detect as many metabolites as possible, thus providing a comprehensive interpretation of the metabolome within a biological entity. The primary steps include the following: adopting a simple and universal preprocessing scheme; thorough metabolomic analysis; extraction of chromatographic peak areas; qualitative identification of chromatographic peaks; statistical analysis to screen for metabolites with significant differences (*p* < 0.05); and the integration of biological knowledge to analyze and interpret the metabolomics results. Untargeted analysis typically utilizes high-resolution mass spectrometry, such as TOF, Orbitrap, and FT-ICR, to detect signal metabolite information in biological samples. This involves accurate identification of metabolites based on precise primary *m*/*z* values, isotope distributions, characteristics, chromatographic retention times, and MS/MS. The conventional LC-MS untargeted approach often employs the data-dependent acquisition (DDA) mode for MS/MS. However, this mode tends to overlook low-abundance metabolites due to its preference for metabolites with strong parent ions, resulting in less than 40% of peaks containing MS/MS information in the total detected. Alternatively, the data-independent acquisition (DIA) mode theoretically achieves comprehensive coverage of metabolite MS/MS information, enhancing the reproducibility and traceability of results. This increases the complexity, as well as the quality, of MS/MS decreases significantly [50], and it is necessary to use deconvolution algorithms to assign MS/MS information to its corresponding parent ions for subsequent data analysis. Open-source deconvolution software reported in the literature includes OpenSWATH [51], MS-DLAL [52], MetDIA [53], DecoMetDIA [54], etc. Currently, untargeted metabolomics is widely used to study the differences in metabolic profiles associated with diseases [55], environmental exposure risks [56], food safety [57], and other fields. However, there are some shortcomings in untargeted metabolomics analysis techniques, such as the narrow linear range of metabolites, insufficient detection sensitivity of low-abundance metabolites, difficult identification of chromatographic peaks, chromatographic peak matching bias, and the cumbersomeness of subsequent data processing.

Targeted metabolomics analysis predominantly focuses on the detection and analysis of specific metabolites, often those involved in specific pathways or belonging to particular categories, achieving high detection sensitivity and quantitative accuracy, as well as a greatly streamlined peak-matching process. This approach has been widely used in the fields of newborn screening for metabolic disorders [58], pharmacokinetics [59], potential biomarkers [60], and the effects of external interventions on metabolic profiles [61]. Targeted metabolomics analyses are often conducted using triple quadrupole (QQQ) mass spectrometry, utilizing either selected ion monitoring (SIM) or multiple reaction monitoring (MRM) modes for metabolite detection. In this approach, metabolite parent ions are first selected in Q1 during a defined retention time; this is followed by collisional fragmentation in Q2; and, finally, specific daughter ions are screened and detected in Q3. This process effectively minimizes interference from other ions, thus greatly enhancing the sensitivity, specificity, linear range, and stability of metabolite detection. However, the targeted approach relies on standards to acquire necessary data, such as retention time, ion pair information, and optimized mass spectrometry parameters. For metabolites without standards and completely unknown metabolites, the method is powerless. Consequently, some researchers have proposed theoretical calculation and prediction strategies to expand the metabolite detection capability of the targeting method. For example, using neutral loss to generate theoretical ion pair lists [62], gangliosides and thioglycolipids are used to generate the theoretical ion pair list [63] based on the structural characteristics of sialic acid ions and sulfate ions. Although this method can improve the problem of insufficient coverage of metabolites for targeted analysis by prediction for some metabolites without standards, it still needs to be implemented based on known fragmentation patterns and related knowledge, and the prediction strategy is not competent for the detection of completely unknown metabolites. In addition, targeted analysis using the parallel reaction monitoring (PRM) acquisition mode of high-resolution mass spectrometry (HRMS) [64] exhibits similar performance to MRM with respect to the quantitative stability of metabolites. However, due to HRMS in the PRM mode capturing information on both primary and corresponding secondary fragmentation ions, it has a distinct advantage in characterizing unknown metabolites. Nevertheless, compared to targeted detection using LRMS, HRMS is limited in terms of metabolite throughput due to slower scanning speeds and slower switching between positive and negative ion modes during the detection process.

Compared to other “omics” studies, metabolomics offers a unique advantage, as it investigates the processes of life at the level of small-molecule metabolites. The focus of metabolomics often lies on intermediates and end products of metabolic pathways, including but not limited to carbohydrates, lipids, and proteins. These metabolites are situated at the endpoint of biological events, reflecting occurrences that have already transpired and are closely related to pathophysiological and functional states. Furthermore, metabolomics facilitates an in-depth exploration of an organism’s metabolic status, analyzing the complex interactions within metabolic networks. Diabetes, a prototypical metabolic disorder, is characterized by significant metabolic dysregulation and numerous associated complications. It has become a significant public health issue, severely impacting national health and imposing substantial socioeconomic burdens. Research into its prevention, early diagnosis, and treatment is thus urgently needed. The ensuing discussion will specifically focus on MS-based metabolomics in diabetes and its complications.

## 3. MS-Based Metabolomics for Diabetes Clinic Research

**Figure 3:**
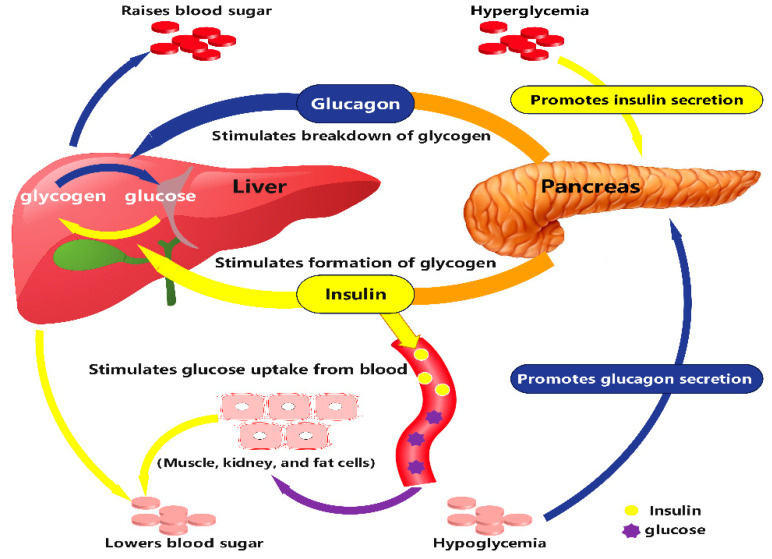
Glucose regulation.

As a typical metabolic disease, diabetes mellitus is characterized by clinical disturbances in glucose (Figure 3) and lipid metabolism. While the oral glucose tolerance test (OGTT) is used as the gold standard for the clinical diagnosis of diabetes [65], its testing process is cumbersome, time-consuming, and laborious, making it unsuitable for rapid screening of large-scale populations. Currently, there is no superior diagnostic or early warning marker for diabetes in clinical practice. Researchers worldwide have been dedicated to identifying diabetes markers with high sensitivity and specificity. Metabolomics technology has proven to be a valuable tool in the study of metabolic disorders caused by diabetes. The metabolic pathways involved in potential biomarkers for diabetes and its complications reflect the intricate metabolic network alterations characteristic of these diseases, encompassing but not limited to the following key pathways and their relationship to diabetes: (1) Insulin signaling and glucose metabolism pathways [66]: The insulin signaling pathway occupies a central role in the pathogenesis of diabetes, where insulin resistance leads to aberrant glucose metabolism, a primary feature of type 2 diabetes mellitus (T2DM). Biomarkers related to this pathway include insulin, C-peptide, and blood glucose levels. (2) Lipid metabolism pathways [67]: Dyslipidemia is common among diabetic patients, involving pathways related to fatty acid β-oxidation, triglyceride synthesis and breakdown, and cholesterol metabolism. Potential biomarkers include plasma triglycerides, low-density lipoprotein (LDL), high-density lipoprotein (HDL) [68], and non-esterified fatty acids (NEFAs). (3) Amino acid metabolism pathways [69]: Studies indicate that certain amino acids, particularly branched-chain amino acids (BCAAs) and aromatic amino acids (AAAs), are aberrantly elevated in diabetic patients, which is linked to decreased insulin sensitivity. Metabolic alterations in these amino acids may serve as early biomarkers for diabetes development. (4) Inflammation and oxidative stress pathways [70]: Inflammation and oxidative stress play critical roles in the progression of diabetes and its complications. Biomarkers such as C-reactive protein (CRP), tumor necrosis factor-alpha (TNF-α), and oxidative stress indicators like malondialdehyde (MDA) may reflect the level of inflammation and oxidative damage within the body [71]. (5) Microvascular complication-related pathways [72]: Microvascular complications, such as diabetic retinopathy, nephropathy, and neuropathy, are closely associated with metabolic pathways related to endothelial dysfunction, angiogenesis, and platelet activation. Potential biomarkers like vascular endothelial growth factor (VEGF) may play significant roles in the development of these complications. Research into these metabolic pathways not only enhances our understanding of the metabolic mechanisms underlying diabetes and its complications but also opens avenues for the discovery of new therapeutic targets and diagnostic criteria. Comprehending the interactions between these metabolic pathways and their biomarkers is crucial for developing personalized medical strategies and improving the prevention, diagnosis, and treatment efficacy of diabetes and its complications. Over the past few years, metabolomics research in diabetes has increased significantly.

### 3.1. MS-Based Research in Diabetes

Metabolomics studies based on mass spectrometry can explore the correlation between metabolites and the risk of the disease, as well as the therapeutic effects of drugs on type 2 diabetes. Yang et al. investigated the anti-type 2 diabetes effect of red ginseng extract using ultra-high-performance liquid chromatography–tandem mass spectrometry (UHPLC-MS/MS) [73], analyzed the blood of rats in both positive and negative ionic modes using non-targeted metabolomics analysis, and employed orthogonal partial least-squares discriminant analysis (OPLS-DA) to further validate the biomarkers and assess the predictive ability of the model. They found that after the intervention with red ginseng extract (RGE), 50 biomarkers showed a tendency to regress and were related to the metabolic pathways of D-arginine and D-ornithine metabolism, D-glutamine and D-glutamate metabolism, taurine and hypotaurine metabolism, Arg biosynthesis, and Trp metabolism. This demonstrated that RGE could effectively modulate metabolites in several pathways associated with T2DM. Zhu et al. established a novel and robust metabolomics platform by integrating field ionization extraction (FIE) with ultra-high resolution Fourier transform ion cyclotron resonance mass spectrometry (FTICR MS) [74]. This platform is used for the analysis of both polar and non-polar metabolite profiles in plasma samples. Moreover, this approach facilitated the identification of potential biomarkers for T2D, and the validation of the method demonstrated good stability and reproducibility. Its high throughput and reproducibility position it as a promising tool for in-depth metabolomics research, enhancing our understanding and diagnostic capabilities in the realm of human diseases. Gall et al. employed UHPLC-MS/MS and GC-MS methodologies to investigate levels of a-hydroxybutyrate (a-HB). Their findings suggest that a-HB can serve as an early biomarker for dysglycemia; monitoring changes in a-HB concentration in fasting human plasma may offer novel perspectives on the early stages of insulin resistance (IR) development and its subsequent transition to T2D [75]. Ho et al. conducted an untargeted metabolomic analysis of human plasma before and after an OGTT experiment and found that most of the metabolites of plasma, including β-hydroxybutyrate, were downregulated and that a small number of metabolites, such as hippurate, were upregulated [76]. Merino et al. employed LC-MS to analyze plasma from 1150 individuals with impaired fasting glucose, identifying alterations in 19 metabolites, including lipids, amino acids, and small-molecule carboxylic acids, which were associated with the development of T2DM [77]. The study further conducted an internal validation of cohorts to assess sensitivity, specificity, and the area under the receiver operating characteristic curve (AUC-ROC). Additionally, ten-fold cross-validation was utilized to validate the biomarkers, ensuring the robustness and reliability of the findings. Thalacker-Mercer et al. used LC-MS to target 16 amino acids in the serum of 124 adult volunteers and found that glycine and insulin resistance had a strong positive correlation and that leucine and isoleucine had a strong negative correlation with insulin resistance as well as T2DM [78]. Menni et al. performed untargeted metabolomic analyses of plasma and urine in females using LC-MS techniques and GC-MS and found that 42 metabolites, including amino acids and sugars, were significantly different between the normal and type 2 diabetes groups. Fourteen metabolites, including amino acids and sugars, were significantly different between normal and impaired fasting glucose groups [79]. The results for urine were validated in an independent cohort and showed good reproducibility. Adams et al. analyzed plasma lipoyl carnitine, free carnitine, and total levocarnitine using LC-MS and found that fatty acids and carnitine matrices combined with this type of carnitine were low in the T2DM population [80]. An untargeted metabolomic analysis of serum from normal and T2DM patients was performed by Zhang et al., who found that dihydrosphingosine, phytosphingosine, and leucine were low and that glycine and lysine were high in the T2DM population [81]. Using UPLC-ESI-Q-TOF-MS technology, untargeted metabolomic analysis of urine from normal and T2DM patients was performed by Zhang et al., who found that acylcarnitines, citric acid, glynine, lysine, and8 metabolites could distinguish the normal and type 2 diabetes groups [82]. Xu et al. employed ultra-performance liquid chromatography coupled with tandem mass spectrometry to examine plasma metabolic profiles in individuals with pancreatic cancer (PC), diabetes mellitus (DM), and healthy controls. The researchers were able to discern distinct metabolite ions between PC and DM patients and healthy subjects. Specifically, lysoPC (20: 4), deoxyadenosine, asparaginyl-histidine, and vaccenyl carnitine concentrations were notably elevated in both the PC and DM cohorts compared to the healthy group. Conversely, phytal, 2 (R)-hydroxydocosanoic acid, behenic acid, catelaidic acid, 2-hydroxyphytanic acid, phytosphingosine, cerebronic acid, docosanamide, and eicosenoic acid levels were significantly diminished in both the PC and DM cohorts [83]. These investigations (refer to Table 2 for additional details) have laid a foundation for elucidating the variations and level changes in metabolite profiles in diabetes while spearheading the quest for potential biomarkers. The identification of markers such as α-hydroxybutyric acid has been instrumental in pioneering early detection methodologies for individuals exhibiting insulin resistance and impaired glucose tolerance within non-diabetic cohorts. Additionally, these biomarkers have been pivotal in tracing the progression from normoglycemia to T2DM. Such research has significantly enriched our comprehension of T2DM metabolic processes and set a robust stage for subsequent scientific inquiries. However, it is pertinent to acknowledge that the limited sample sizes and the representativeness of the study populations might constrain the broader applicability of these insights. Given the intricate nature of metabolic pathways and interindividual biological variability, future research endeavors necessitate more rigorously controlled trials encompassing diverse population samples to affirm the clinical relevance of these emerging potential biomarkers.

In recent years, researchers have begun to explore the use of volatile organic compounds (VOCs) in respiratory gases as biomarkers for the diagnosis of diabetes mellitus. Wang et al. investigated the use of breath testing in the diagnosis of diabetes mellitus through a systematic review and meta-analysis. The results showed that the isotope CO2 is the most commonly used breath test biomarker with high sensitivity and specificity [84]. It can be detected by techniques such as GC-MS, which provides a new non-invasive method for the early diagnosis of diabetes and helps to improve the efficiency and accuracy of diabetes diagnosis. Trefz et al. employed direct real-time proton transfer reaction time-of-flight mass spectrometry (PTR-TOF-MS) for breath analysis to detect volatile organic compounds (VOCs) in pediatric patients with type 1 diabetes mellitus (T1DM). They observed significant changes in the concentrations of acetone, 2-propanol, pentanal, ethanol, dimethyl sulfide, isoprene, and limonene over the course of the nine-hour examination period, which could be linked to metabolic processes [85]. Jiang et al. utilized a real-time ringdown breath acetone analyzer based on cavity ringdown spectroscopy (CRDS) to detect acetone concentrations in breath samples and compared it to a certified GC-MS facility for validation. The results demonstrated that the acetone concentrations obtained by both methods were consistent, with a linear fitting coefficient of 0.98 [86]. This confirmed the reliability and accuracy of the ringdown breath acetone analyzer. The study concluded that the analyzer could be employed for potential diabetic screening and management by measuring breath samples. However, there is still a need for standardization before clinical practice, including subject control, breath sampling, and optimization of analytical methods.

### 3.2. MS-Based Research in Gestational Diabetes Mellitus

Gestational diabetes mellitus (GDM) is one of the most common metabolic disorders in pregnancy, usually occurring in the 3rd–6th or 6th–9th month of pregnancy [87]. Over the past few decades, the prevalence of GDM has increased worldwide (including China) [88], with prevalence rates ranging from 9.3% to 25.5% globally [89]. Early diagnosis of GDM and meticulous management of blood glucose levels are essential measures to significantly mitigate complications for both the mother and the infant [90]. Currently, GDM is mainly screened by detecting blood glucose changes before and after sugar consumption in pregnant women between 24 and 28 weeks of pregnancy, but this method diagnoses patients relatively late, which has seriously jeopardized the health of mothers and infants. Therefore, the search for new early biomarkers is particularly important. Burzynska-Pedziwiatr et al. used HPLC-MS/MS for targeted metabolomics [91] to analyze peripheral blood in cases of gestational diabetes mellitus in the middle and postnatal trimesters and after one year. The results of the study showed that plasma concentrations of metabolites such as arginine (Arg), glutamine (Gln), histidine (His), methionine (Met), phenylalanine (Phe), and serine (Ser) were significantly decreased in patients with gestational diabetes mellitus and that arginine was useful to accurately differentiate between GDM and normoglycemia (NGT) in the development of GDM, as well as in the early detection of GDM, and that it also helped to predict increased risk of T2DM in women. Zhang et al. employed UPLC-MS/MS to concurrently quantify 13 estrogens [92], including estrone (E1), estradiol (E2), and estriol (E3), as well as their hydroxylated and methylated metabolites, in urine samples of gestational diabetes mellitus (GDM) subjects. Their findings revealed elevated urinary levels of E1, E2, and the majority of estrogen metabolites in pregnant women with GDM compared to levels in healthy pregnant counterparts. Notably, the mean concentration of 2-hydroxyestrone (2-OHE 1) in GDM subjects was 13.2 times lower than that observed in healthy pregnant women. Significant disparities in the predominant urinary estrogen species were noted between the GDM group and the normal pregnant cohort, with 2-methoxyestrone (2 MeOE 1) and E3 being the highest species. A comparative analysis was conducted on blood samples from 34 pregnant women with GDM and 34 healthy pregnant controls, matched by gestational weeks, using LC-MS in both positive and negative ion modes [93]. Significant differences were observed in the content and biosynthesis of metabolites related to unsaturated fatty acid biosynthesis, phenylpropanoid biosynthesis, the carbon fixation pathway in prokaryotes, terpenoid and steroid biosynthesis, the two-component system, ascorbic acid and glyoxylate metabolism, and furfural degradation between the GDM and control groups. The method’s stability and reproducibility were demonstrated by the area under the receiver operating characteristic (ROC) curve (AUC values < 0.882), validating new biomarkers for GDM. The research sheds light on the complex metabolic alterations that occur during pregnancy. Studies focusing on sphingolipids enhance our understanding of the maternal metabolome and lipidome, providing deeper insights into the physiological processes during pregnancy. The use of metabolomics analysis combined with targeted quantitative analysis represents an advancement in research methodology. This dual approach allows for a more nuanced understanding of specific lipid changes during pregnancy, contributing to the precision and depth of metabolic studies in obstetrics and gynecology. The identification of potential biomarkers and other related studies [94,95] are shown in Table 2.

Although biomarkers are significant for metabolomics studies, their validation is necessary in diabetes metabolomics research to ensure reliability and clinical utility. Validation methods typically involve independent cohort validation, cross-validation, and external validation. Independent cohort validation involves assessing the performance of potential biomarkers in cohorts or datasets entirely different from the original study population. This validation method ensures that a biomarker demonstrates similar predictive performance even when validated on independent cohorts [96] distinct from the original study population [79]. Independent cohort validation is crucial for evaluating the generalizability and applicability of biomarkers across diverse populations. Cross-validation entails partitioning a dataset into multiple subsets, typically a training set and a validation set, and iteratively performing model training and validation [97]. Common cross-validation techniques include k-fold cross-validation, where the dataset is divided into k subsets, with each subset taking a turn as the validation set, while the remaining subsets are used for training. By repeating this process multiple times, the performance metrics of a biomarker are averaged, providing an assessment of its performance within the same dataset [98]. External validation refers to the validation of biomarkers on datasets that are entirely independent of the original study dataset. This validation method is commonly employed to evaluate the generalizability and robustness of biomarker findings across different datasets or populations. External validation can be conducted using datasets from other laboratories, different geographical regions, or distinct populations, ensuring the reliability and applicability of the biomarkers. Commonly used biomarker validation methods can be categorized based on the source and nature of the validation dataset [99]. Both independent cohort validation and external validation involve validation on different datasets, aimed at assessing the generalizability of biomarkers. In contrast, cross-validation is performed within the same dataset, evaluating biomarker performance on the given dataset.

**Table 2:** Summary of important findings and metabolites of diabetes and its complications.

Disease	Objective	Methods	Sample Source	Major Findings	Levels of Metabolites	Reference
**Diabetes mellitus** **(DM)**	Untargeted metabolomics analysis of anti-diabetic effects	UHPLC-MS/MS	Rat serum samples	Red ginseng extract intervention regulated 50 biomarkers, involving multiple metabolic pathways, including amino acid metabolism, glycerol–phospholipid metabolism, and fatty acid metabolism 5-HTP should be used as a potential lead compound	MOD: Phe↓, TUDCA↓, LysoPCs↓, LysoPEs↓, 2-methylbutyroylcarnitine↓, Gamma-tocotrienol↓, equo l4′-O-glucuronide↓	[73]
	Plasma metabolomics applied to type 2 diabetes research	FIE -FTICR MS	Mouse plasma samples	Successfully detected over 300 statistically significant metabolic features, identifying novel T2DM biomarker candidates	Among the non-polar metabolites: lipids with shorter acyl chains↑, myristic↑, palmitic↑, arachidonic acids↑, PCs of fewer total carbons↓, PCs with more total carbons↑, hexoses↑	[74]
	Searching early marker for dysglycemia	UHPLC-MS/MS, GC-MS	Human plasma samples	Identified α-hydroxybutyric acid as a potential biomarker for both insulin resistance and impaired glucose regulation	Insulin resistant subjects: a-HB↑, 1-linoleoyl-GPC↓, glycine↓, 3-methyl-2-oxobutyrate↑, 1-oleoyl-GPC↓, creatine↑, decanoylcarnitine↓, octanylcarnitine↓, 1-stearoyl-GPC↓, adrenate (22:4n6)↑, stearate↑, 1-palmitoyl-GPC↓, palmitate (16:0)↑, margarate↑	[75]
	Metabolite profiles during oral glucose challenge	LC-MS/MS	Human plasma samples	91 out of 110 metabolites significantly changed with OGTT Downregulation of metabolites like β-hydroxybutyrate and upregulation of metabolites like hippurate observed	Metabolite changes following OGTT in insulin-resistant patients: b-Hydroxybutyrate↑, Isoleucine↑, Lactate↓, Orotate↓, Pyridoxate↑	[76]
	Metabolomics insights into early type 2 diabetes pathogenesis	LC-MS/MS	Human plasma samples	Discovered changes in 19 metabolites, including lipids, amino acids, and small organic acids, associated with the onset of type 2 diabetes	Type 2 diabetes: glycine↑, taurine↑, phenylalanine↑	[77]
	To examine the relationships between amino acid levels and the hyperinsulinemic–euglycemic clamp	LC-MS	Human serum samples	Found positive correlation of glycine with insulin resistance and negative correlation of leucine and isoleucine with insulin resistance and type 2 diabetes	T2DM: Gly↓, Leu/Ile↑, Val↑, His↓, Asx↑, Glx↑	[78]
	Searching for novel molecular markers that arise before and after hyperglycemia	LC-MS, GC-MS	Women’s plasma and urine samples	Detected significant differences in 42 metabolites, including amino acids and sugars, between normal and type 2 diabetes groups, as well as in 14 metabolites between normal and impaired fasting glucose groups	IFG and subjects with T2DM: Valine↑, Isoleucine↑, Leucine↑, 3-methyl-2-oxovalerate↑, 4-methyl-2-oxopentanoate↑, 3-methyl-2-oxobutyrate↑	[79]
	Plasma acylcarnitine profiling in T2D	LC-MS	Women’s plasma samples	Discovered decreased levels of acylcarnitines (fatty acid derivatives) and increased levels of amino acids such as glycine and lysine in type 2 diabetes patients	T2DM: Calculated total acylcarnitines (total free)↑, Acyl/free ratio↑, C2↑, C3↓, C6↑, C8↑, C10↑, C14↑, C18:1↑, C8-dicarb↑, summed C10-C14 acylcarnitines↑, total acylcarnitines↑	[80]
	UPLC-oaTOF-MS for serum profiling in diabetic patients	UPLC-oaTOF-MS	Human serum samples	The metabolomics based on UPLC–oaTOF-MS could reflect the balance of homeostasis and metabolism of nourishment	T2DM: Phytosphingosine↓, Dihydrosphingosine↓, Leucine↓	[81]
	Determine the differences in metabolite concentrations between T2D patients and healthy volunteers	UPLC-ESI-Q-TOF-MS	Human urine samples	Identified 12 metabolites, including acylcarnitines, citric acid, canine urea, and taurine, distinguishing between normal and type 2 diabetes groups	T2DM: Adiponectin↑, Acylcarnitines↑, Citric acid↓, Kynurenic acid↓, 3-Indoxyl sulfate↑, bile acids↑, Urate, glucose↑, Glycine↑, Glucuronolactone↓, Lysine↓, Phosphate↓	[82]
	Identify biomarker signatures to differentiate pancreatic cancer from type 2 diabetes mellitus in early diagnosis	UPLC-MS/MS	Human plasma samples	Successful screening of differential metabolite ions between pancreatic cancer and DM patients and healthy individuals	DM:LysoPC (20: 4)↑, Deoxyadenosine↑, Asparaginyl-histidine↑, Vaccenyl carnitine↑, Phytal↓, 2 (R)-hydroxydocosanoic acid↓, Behenic acid↓, Catelaidic acid↓, 2-hydroxyphytanic acid↓, Phytosphingosine↓, Cerebronic acid↓, Docosanamide↓, Eicosenoic acid↓	[83]
**Gestational diabetes mellitus** **(GDM)**	Identify early risk indicators for GDM	LC-MS/MS	Women’s peripheral blood	Arginine assists in distinguishing GDM from NGT, early detection of GDM, and predicting increased risk of T2DM in women	GDM group: Arg↓, Gln↓, His↓, Met↓, Phe↓, Ser	[91]
	Investigate estrogen metabolism imbalance in GDM	UPLC-MS/MS	GDM women’s urine samples	Successfully detected and quantified thirteen estrogens in different samples	GDM group: E1↑, E2↑, E3↓, 16epiE3↑, 17epiE3↑, 16α-OHE1↑, 2-OHE2↑, 2MeOE1↑, 4MeOE1↓, 2MeOE2↑, 4MeOE2↓, 2-OHE1↓, 4-OHE1↑	[92]
	Unique biomarker characteristics in gestational diabetes mellitus	LC-MS	Human urine samples	184 metabolites were increased and 86 metabolites were decreased in the positive ion mode, and 65 metabolites were increased and 71 were decreased in the negative ion mode	GDM group: Eicosapentaenoic Acid↓; Docosahexaenoic Acid↑; Docosapentaenoic Acid; Arachidonic Acid↑; α-Ketoglutaric Acid↑; Phosphoric Acid↑; Citric Acid↑; Genistein↓; Daidzein↓; 2-Furoic Acid↑	[93]
	Metabolic alteration of circulating steroid hormones in women with gestational diabetes mellitus	UPLC-MS/MS	Human urine samples	16OHE1 may be a strong marker associated with the risk for GDM	GDM group: 16OHE1↑; E1-G/S↑	[94]
	Effects of pregnancy on plasma sphingolipids using metabolomics	LC/MS/MS	Women’s blood samples	A wide range of sphingolipids have altered plasma concentrations during pregnancy compared to postpartum, including ceramides, sphingomyelins, and sphingosines	During pregnancy, the most altered metabolite of interest was sphingomyelin (d18:1/20:2, d18:2/20:1, d16:1/22:2)↑	[95]
Diabetic cardiomyopathy (DCM)	Multi-omics of a preclinical model of diabetic cardiomyopathy	LC-MS/MS	Rat blood and ventricle samples	Metabolomics detected 19 amino acids, of which 12 were significantly altered	High-fat diet with STZ group: Arg↓, Asn↓, Asp↓, Gln↓, Gly↓, His↓, Met↓, Ser↓, Glu↑, Val↑	[100]
	To distinguish T2DM patients with or without damp-heat syndrome (DHS) and discover biomarkers	UPLC-TOFMS/MS	Human plasma samples	Vitamin and amino acid metabolism were changed in T2DM patients with the syndrome of DHS	DHS: imidazole↓, L-pipecolic acid↓, L-citrulline↓, L-carnitine↓, 3′-O-methylguanosine↓, pantothenate↑, sphingomyelin↑, thioetheramide-PC↑	[101]
	Metabolic markers in patients with chronic heart failure before and after LVAD implantation	LC-MS/MS	Human plasma samples	Some acylcarnitines were more prominently altered in ICM or DCM when compared to the control, which could be interesting for the specific metabolic characterization of ICM and DCM	LVAD implantation postoperatively: SM (OH) C14:1↑, SM (OH) C22:1↑, SM C16:0↑, and SM C24:0↑ Potential prognostic markers: lysoPCs, proline (Pro)	[102]
	To investigate the differences in circulating LCAC levels in HF patients with and without DM	MS	Human plasma samples	LCAC biomarkers were associated with exercise status and clinical outcomes differentially in HF patients	DCM group: LCACs (C16, C16:1, C18, C18:1, C18:2)↑	[103]
	Metabolic profiling, mitochondrial dysfunction, ob/ob mouse heart	LC-MS	Mouse heart tissue	There was an age-dependent decrease in myocardial acylcarnitine concentrations in ob/ob mice fed either RCD or HFD compared with those in the corresponding WT mice	HFD ob/ob mouse: myocardial acylcarnitine↓	[104]
Diabetic encephalopathy (DE)	Identify hippocampal metabolic alterations in a rat model of diabetic encephalopathy induced by STZ	GC-MS	Rat hippocampus Samples	Lower levels of NAA and DHAP and higher levels of homocysteine and glutamate in DE group rats, indicating a potential correlation between cognitive impairment and these metabolic changes	DM group: NAA↓, DHAP↓; homocysteine↑, glutamate↑	[105]
	To test the hypothesis that brain glycogen metabolism is impaired in type 2 diabetes	GC-MS	Rat cortex, hippocampus, striatum, and hypothalamus samples	Impaired brain glycogen metabolism related to T2D, suggesting a connection between brain glycogen metabolism and type 2 diabetes	The phosphorylation rate of glycogen synthase was increased	[106]
Diabetic nephropathy (DN)	Plasma esterified and non-esterified fatty acid metabolic profiling in diabetic nephropathy	GC-MS	Human plasma samples	Developed a new method for simultaneous identification of 25 NEFAs and EFAs	Control–DM: EFAs↓, NEFAs↑; DM-DNШ: EFAs↑	[107]
	UPLC-oaTOF-MS for serum profiling in diabetic patients	UPLC-oaTOF-MS	Human serum samples	The metabolomics based on UPLC–oaTOF-MS could reflect the balance of homeostasis and metabolism of nourishment	DN group: Phytosphingosine↓, Dihydrosphingosine↓, Leucine↓	[81]
	Identification of potential serum metabolic biomarkers of diabetic kidney disease	UPLC-ESI-MS/MS	Human serum samples	Identified 11 new metabolites closely related to DKD through comprehensive targeted metabolomic profiling. Provides insights into various early metabolic signs of DKD, aiding prediction and prevention in populations	Hexadecanoic Acid (C16:0)↑, Linolelaidic Acid (C18:2N6T)↑, Linoleic Acid (C18:2N6C)↑, Trans-4-Hydroxy-L-Proline↓, Aminocaproic Acid↓, L-Dihydroorotic Acid↓, Methylmercaptopurine↓, Piperidine↓, Azoxystrobin Acid↑, Lysopc 20:4↑, Cuminaldehyde↓	[108]
Diabetic peripheral neuropathy (DPN)	To examine the serum lipidomic profile associated with neuropathy in type 2 diabetes	MS	Human serum samples	Circulating acylcarnitines, free fatty acids, phosphatidylcholines, and lysophosphatidylcholines are associated with neuropathy status in type 2 diabetes	Pima participants with T2D: medium-chain acylcarnitines↓, total free fatty acids↑, phosphatidylcholines↓, lysophosphatidylcholines↑	[109]
	To investigate the neuroprotective effect of Jin-Mai-Tong (JMT) decoction on diabetic rats with peripheral neuropathy and to elucidate the potential mechanism	UPLC/QTOF-MS	Rat serum samples	21 metabolites were identified; JMT decoction has an obvious protective effect against DPN; lipid metabolism, TCA cycle, amino acid metabolism	STZ group: 2-Ketobutyric acid↑, Paraxanthine↑, Leucyl-Cysteine↑, Artonin K↑, Deoxycytidine↑, Oxalacetic acid↑, LysoPC (18:3)↑, LysoPE (0:0/18:2)↑, Lysophosphatidic acid (0:0/18:2)↑, Delcorine↑, LysoPE (0:0/22:6)↑, Hexadec-2-enoyl carnitine↑, LysoPE (0:0/16:0)↑Lithocholic acid glycine conjugate↑, N-(1-Deoxy-1fructosyl) leucine↑, Stearoylcarnitine↑, Glycerol tripropanoate↑, Retinyl beta-glucuronide↑, C46H74NO10P↑, Hexyl dodecanoate↑, Tyr-Pro-Phe↓	[110]
	Investigate the effects of Tang Luo Ning (TLN) on DPN in rats using an LC-MS metabolomics approach	HPLC-IT-TOF/MS	Rat serum sample	14 potential biomarkers; TLN could improve the peripheral nerve function and reduce the demyelination of the sciatic nerve in DPN rats; TCA cycle; glycine, serine, and threonine metabolism; glyoxylate and dicarboxylate metabolism	MOD: 3-Butenoic acid↓, Acetylcarnitine↓, Citrate↑, Creatine↑, Creatinine↓, Fumarate↑, Glyceric acid↓, Glycine↑, Lactate↓, LysoPC (22:5)↑, Palmitoyl glucuronide↑, Riboflavin↓, Succinate↓, Tryptophan↓	[111]
Diabetic foot ulcers (DFUs)	To evaluate and identify specific amino acids associated with the healing outcomes of patients with DFUs	LC-MS/MS	Human blood samples	Higher levels of serum arginine, isoleucine, leucine, and serine were observed in the healed ulcer group compared to the non-healing group, indicating potential biomarkers for wound healing in DFUs	DFUs: Arginine↑, Leucine↑, Isoleucine↑, Threonine↑	[112]
Diabetic retinopathy (DR)	To identify tear fluid biomarkers for differentiating between PDR and NPDR in T2D patients	GC-MS	Human tear samples	D-Glutamine and D-glutamate metabolism was significantly highlighted in the PDR group as compared to the non-diabetic group. The metabolites in tears could be potential biomarkers in DR analysis	PDR group: Guanosine↑, Uric acid↑, D-(+)-malic acid↑, Pimelic acid↑, Azelaic acid↑, 2-Hydroxybenzothiazole↑, N, N-Diethyl-4-methoxybenzamide↓, Homovanillic acid↓, Phenol↓, Pipecolic acid↓, Guvacoline↓, Spironolactone↓, N,N-Diethyl-3-methoxybenzamide↓, Diazepam↓, Prostaglandin F2α 1-11-lactone↓, 2-Methoxyestrone↓, Tretinoin↓	[113]
	Identify a specific plasma metabolic profile associated with DR as distinct from diabetes alone	GC-MS	Human plasma samples	Elevated levels of 2-deoxyribonucleic acid, 3,4-dihydroxybutyric acid, erythritol, gluconic acid, and ribose were found in patients. These were validated in an independent sample set and considered as potential biomarkers for diabetic eye disease	DR group: 2-deoxyribonucleic acid↑, 3,4-dihydroxybutyric acid, erythritol↑, gluconic acid↑, ribose↑	[114]
	Identify serum metabolite biomarkers for DR using various metabolomics platforms	LC-MS, GC-MS	Human serum samples	Identified 348 metabolites with significant differences between groups. 12-Hydroxyeicosatetraenoic acid and 2-pyrrolidinone were significantly elevated in patients with diabetic eye disease	DR group: 12-Hydroxyeicosatetraenoic acid↑, 2-pyrrolidino↑ne	[115]

DM, diabetes mellitus; T2D or T2DM, type 2 diabetes mellitus; GC-MS, gas chromatography–mass spectrometry; GC-MS/MS, gas chromatography–tandem mass spectrometry; LC-MS, liquid chromatography–mass spectrometry; LC-MS/MS, liquid chromatography–tandem mass spectrometry; ultra-performance liquid chromatography coupled to electrospray ionization quadrupole time-of-flight mass spectrometry (UPLC-ESI-Q-TOF-MS/MS); UHPLC-MS/MS, ultra-high-performance liquid chromatography–tandem mass spectrometry; DKD, diabetic kidney disease; PDR, proliferative diabetic retinopathy; NPDR, non-proliferative diabetic retinopathy; MOD, model group; 5-HTP, 5-hydroxytryptophan acid; TUDCA, Tauroursodeoxycholic acid; Phe, phenylalanine; LysoPCs, Lysophosphatidylcholines; LysoPEs, Lysophosphatidylethanolamines; FIE-FTICR MS, field ionization extraction with ultra-high resolution Fourier transform ion cyclotron resonance mass spectrometry; a-HB, a-hydroxybutyrate; GPC, glycerophosphocholine; OGTT, oral glucose tolerance test; IFG, impaired fasting glucose; Gly, Glycine; Leu/Ile, leucyl-isoleucine; Val, Valine; His, histidine; Asx, aspartate/asparagines; Glx, glutamate/glutamine; C2, Acetylcarnitine; C3, Propionylcarnitine; C6, Hexanoylcarnitine; C8, Octanoylcarnitine; C10, Decanoylcarnitine; C14, Myristoylcarnitine, C18:1, Oleoylcarnitine; C8-dicarb, Suberoylcarnitine; Arg, arginine; Gln, glutamine; Met, methionine; Ser, serine; E1, estrone; E2, estradiol; E3, estriol; 16epiE3, 16-epiestriol; 17epiE3, 17-epiestriol; 16α-OHE1, 16α-hydroxyestrone; 2-OHE2, 2-hydroxyestradiol; 2MeOE1, 2-methoxyestrone; 4MeOE1, 4-methoxyestrone; 2MeOE2, 2-methoxyestradiol; 4MeOE2, 4-methoxyestradiol; 2-OHE1, 2-hydroxyestrone; 4-OHE1, 4-hydroxyestrone. GDM, gestational diabetes mellitus; TCA, tricarboxylic acid; E1-G/S, Estrone-Glucuronide/Sulfate; Asn, asparagine; Asp, aspartic acid; LVADs, left ventricular assist devices; ICM, ischemic cardiomyopathy; LCAC, long-chain acylcarnitines; HF, heart failure; RCD, regular chow diet; HFD, high-fat diet; NAA, N-acetyl aspartate; DHAP, dihydroxyacetone phosphate; NEFAs, non-esterified fatty acids; EFAs, esterified fatty acids.

## 4. MS-Based Metabolomics in Clinical Cases of Diabetes-Induced Complications

### 4.1. MS-Based Research in Diabetic Cardiomyopathy

While diabetes is related to various clinical complications, cardiovascular diseases contribute to approximately 65% of diabetes-related mortality [116], and T2D patients have a 2~5-fold higher risk of developing CVD and also very commonly diabetic cardiomyopathy (DCM) [117]. DCM represents a unique myocardial structural abnormality accompanied by functional impairment in patients with diabetes, yet its pathogenesis remains not fully elucidated. In a study conducted by Li et al., sequential and integrated analysis of the proteome, lipidome, and metabolome was performed using liquid chromatography–tandem mass spectrometry [100]. They found that in T2DM the heart redirects excess acetyl-CoA towards ketogenesis and incomplete β-oxidation through the formation of short-chain acylcarnitine, potentially contributing to increased cardiovascular disease risk in T2DM patients. The research aimed to distinguish between T2DM patients with or without damp-heat syndrome (DHS). Two groups of T2DM patients, one with damp-heat syndrome and the other without, were diagnosed using the dialectical diagnosis approach of traditional Chinese and Western medicine, with 30 cases in each group. Shao et al. [101] utilized the LC-MS/MS technique to analyze harvested plasma samples, identifying 22 differentially abundant metabolites and 14 syndrome-related biomarkers. The discovery of these syndrome-related biomarkers holds particular significance for personalized treatment approaches. Biomarkers associated with traditional Chinese medicine (TCM) syndromes can aid in clinical diagnosis, contribute to the modernization of TCM, and provide valuable insights into the pathogenesis of diabetes. Hilse et al. [102] employed the LC-MS/MS method to analyze changes in metabolic markers before and after left ventricular assist device (LVAD) implantation in patients with chronic heart failure (HF). The study involved analyzing plasma metabolites from 20 patients with ischemic cardiomyopathy (ICM), 20 patients with dilated cardiomyopathy (DCM), and 20 healthy controls. The results revealed that 63 out of the 188 measured metabolites changed in HF patients before and after LVAD implantation. Interestingly, only three metabolites returned to their pre-LVAD concentrations 100 days after the implantation. Furthermore, the pre-LVAD differences between DCM and ICM were primarily related to amino acids and biogenic amines. These findings indicate that LVAD implantation can reverse abnormal metabolite levels in HF patients. Other studies on diabetic myocardial metabolomics [103,104] are shown in Table 2. Validation methods across these studies typically involved statistical and analytical techniques, such as ROC curve analysis, multivariate analysis (e.g., OPLS-DA), and comparison of metabolite concentrations pre- and postintervention (e.g., LVAD implantation). These methods aimed to assess the diagnostic accuracy, specificity, sensitivity, and potential of identified metabolites as biomarkers for various conditions (e.g., GDM and heart failure). The combined use of metabolic profiling, statistical validation, and clinical correlation was central to validating new metabolic biomarkers across these studies. These studies collectively advance the understanding of diabetic cardiomyopathy by focusing on metabolic dysfunctions and biomarkers. Each study contributes uniquely, offering insights into specific metabolic pathways and potential biomarkers, such as long-chain acylcarnitines and differentially abundant metabolites, in T2DM patients. These studies are complementary, as they collectively provide a multi-dimensional view of the metabolic changes in diabetic cardiomyopathy, highlighting the complex interplay between metabolic dysfunctions, diabetes, and heart health. This comprehensive approach is advantageous in developing a more holistic understanding and potential therapeutic targets for diabetic cardiomyopathy.

### 4.2. MS-Based Research in Diabetic Encephalopathy

Chronic hyperglycemia in diabetic patients can result in a range of macrovascular and microvascular complications. Due to the high consumption of oxygen and glucose by diabetes, the brain is also affected [118]. It is estimated that approximately 40% of diabetic patients will experience mild to moderate cognitive dysfunction, a condition known as diabetic encephalopathy (DE). Clinical studies have shown that diabetic patients with DE are at risk of progressing to dementia [119], so elucidating the molecular pathophysiological changes underlying the onset and progression of DE is vital for its prevention, diagnosis, and treatment [120]. As a complex metabolic disorder, DE is intricately linked to the aberrant metabolism of small-molecule biological compounds [105]. Chen et al. explored metabolic changes in the hippocampus of a rat model with cognitive dysfunction induced by streptozotocin (STZ) using GC-MS technology [105]. The study revealed lower levels of N-acetyl aspartate (NAA) and dihydroxyacetone phosphate (DHAP) in the DE group compared to the normal group. Additionally, the levels of homocysteine and glutamate were higher in the DE group, indicating cognitive dysfunction. These findings suggest that these compounds could potentially serve as potential biomarkers for DE. Soares et al. [106] employed gas chromatography-mass spectrometry to quantify local 13C enrichment fractions of glucose and glycogen in the cortex, hippocampus, striatum, and hypothalamus, revealing an association between impaired brain glycogen metabolism and T2D. Multivariate statistical analyses, such as PCA and OPLS-DA, played a crucial role in validating the identified biomarkers by demonstrating significant metabolic differences between diabetic and control groups. Refer to Table 2 for additional details. These studies utilize animal models to investigate glycogen metabolism and hippocampal metabolic changes in diabetes, providing valuable insights into the biochemical pathways affected by the disease. However, the primary limitation is their reliance on specific animal models, which may not entirely mimic the human condition.

### 4.3. MS-Based Research in Diabetic Nephropathy

Diabetic nephropathy (DN), a severe complication of diabetes mellitus, is often considered irreversible in modern medicine. Progression to advanced renal failure is nearly inevitable for patients with diabetic nephropathy, posing a significant threat to life and health. Therefore, early diagnosis and slowing down the progression of diabetic nephropathy are of paramount importance. Diabetic nephropathy can be classified into five stages based on Mogensen’s pathological staging criteria. However, due to the invasive nature of pathological diagnosis, it is often employed only when necessary and is not suitable for routine clinical screening. Currently, the clinical diagnostic model that uses clinical proteinuria as the gold standard can only identify patients with stage III diabetic nephropathy. For patients in stages I and II, who do not exhibit significant clinical proteinuria, there is no current method to distinguish them from the general diabetic population in clinical screenings. This limitation results in missed opportunities for early intervention in diabetic nephropathy patients. By establishing a variety of chromatography–mass spectrometry coupled techniques, it is feasible to qualitatively detect several thousand molecular ions in serum metabolic fingerprint spectra and quantitatively analyze nearly a hundred targeted metabolites. This approach holds promise for the early diagnosis of stages I and II diabetic nephropathy. Researchers have developed a novel metabolic method for non-esterified fatty acids (NEFAs) and esterified fatty acids (EFAs) in plasma using GC-MS [107]. With just 10 μL of plasma, this technique can simultaneously identify 25 types of FAs, which can be utilized to infer the pathological relationship between FA levels and DM as well as DN. Zhang et al. [81] applied UPLC-oaTOFMS to analyze the total serum profiles of 8 patients with DN, 33 patients with T2DM, and 25 healthy volunteers and demonstrated that disturbances in amino acid metabolism and phospholipid metabolism existed in patients with diabetic nephropathy. Zhang et al. [108] conducted a broadly targeted metabolomics study using UPLC-MS/MS to analyze serum metabolites and identified a total of 11 new metabolites closely related to diabetic kidney disease (DKD). The study suggests various early metabolic signs of DKD which can aid in predicting and preventing DKD in the population. (Refer to Table 2 for additional details.) The studies collectively explore the metabolic alterations in DKD and T2DM, employing different analytical methods to detect various metabolites and fatty acids. While each study identifies unique potential biomarkers, together they highlight the metabolic complexity in DKD and T2DM. Across these studies, advanced analytical and statistical methods, including UPLC-oaTOF-MS and GC-MS, are utilized to profile and validate new metabolic biomarkers for DN, DM, and related complications. These approaches enable the identification of disease-specific metabolite alterations, offering insights into the metabolic pathways involved in disease pathogenesis. The complementary findings suggest a multifaceted approach to understanding and diagnosing these conditions, emphasizing the need for integrated metabolic profiling in medical research.

### 4.4. MS-Based Research in Diabetic Peripheral Neuropathy

Diabetic peripheral neuropathy (DPN) is the most common type of diabetic neuropathy, which can manifest as distal sensory deficits or neuropathic pain, and is a major cause of foot ulcers, non-traumatic amputations [121], and increased morbidity and mortality in diabetic patients [122]. Previous studies have identified several factors associated with the formation of diabetic peripheral neuropathy (DPN), including polyol bypass activation, protein non-enzymatic glycosylation, oxidative stress, inflammation, and neurotrophic disorders. However, the specific pathophysiological mechanisms remain unclear. Recent metabolomics studies have delved into metabolic disorders, pathological mechanisms, and biological markers of DPN, shedding more light on the subject [123]. Afshinnia et al. performed quantitative mass spectrometry of serum lipids (435 species from 18 classes) in 69 patients with type 2 diabetes mellitus [109]. The results demonstrated a significant decrease in serum chain acylcarnitines and an increase in total free fatty acids at baseline, independent of chain length and saturation, which were associated with peripheral neuropathy occurring at follow-up. Zhang et al. [110] explored the neuroprotective effects of Jin-Mai-Tong (JMT) in diabetic peripheral neuropathy rats from a metabolomic perspective by subjecting the collected serum samples to UPLC-based/QTOFMS and multivariate statistics for untargeted metabolomics analysis. The metabolomics study revealed significant changes in the serum metabolic profiles of the model and control groups. A total of 21 metabolites were recognized as potential biomarkers linked to the therapeutic effects of JMT tonic. Sixteen of these potential biomarkers were identified in both the JMT high-dose (JMT-H) and JMT low-dose (JMT-L) treatment groups, while the other five potential biomarkers were identified only in the JMT-H group. These metabolites predominantly participated in lipid metabolism, the tricarboxylic acid cycle, and amino acid metabolism, among other pathways. Correlation analysis indicated a negative relationship between mechanical pain threshold and distal nerve fiber density with metabolites associated with lipid metabolism and the tricarboxylic acid cycle. It was concluded that the JMT decoction has obvious protective effects on diabetic peripheral neuropathy rats, and its mechanism may be related to improving the metabolic disorders in peripheral neuropathy rats. Li et al. [111] used the HPLC-IT-TOF/MS technique to study the mechanism of action of Tang Luo Ning (TLN) on high-glucose-induced diabetic peripheral neuropathy, and metabolic pathway analysis was used to explore the effects of DPN and TLN on the metabolism of rats. Metabolomics analysis revealed 14 potential biomarkers (citrate, creatine, fumarate, glycerate, glycine, succinate, etc.) of the effects of DPN and TLN treatment. Prolonged hyperglycemia can cause metabolic abnormalities in several pathways, which are closely associated with structural damage and functional changes in the nervous system. (Refer to Table 2 for additional details.) These articles offer new insights in the following areas: 1. they provide evidence on the effectiveness of traditional Chinese medicines in treating diabetic neuropathy, expanding the therapeutic options and understanding of alternative medicine in this field; 2. they used metabolomic and lipidomic profiling to understand the underlying mechanisms of diabetic neuropathy and the impact of treatments; 3. they contribute to the identification of potential biomarkers that could be used for early diagnosis or predicting the progression of diabetic neuropathy.

### 4.5. MS-Based Research in Diabetic Foot Ulcers

Diabetic foot ulcers (DFUs) are significant complications of diabetes, contributing to disability and mortality. Around 15–25% of individuals with diabetes develop DFUs, making them a leading cause of morbidity and mortality. Patients with DFUs face a 2.5 times higher risk of death within 5 years compared to diabetic patients without ulcers [124]. Early access to risk stratification for DFUs in diabetic patients can reduce hospitalization, disability, and mortality rates. Hung et al. [112] analyzed blood samples from 57 DFU patients using targeted metabolomics with LC-MS/MS; this study aimed to evaluate specific amino acids associated with wound healing outcomes in patients with DFUs. Among these ulcers, 19 were non-healing and 38 were healing. The healing group exhibited significantly higher serum levels of arginine, isoleucine, leucine, and threonine compared to the non-healing group. This finding highlights the potential significance of these potential biomarkers in the healing process of DFUs. Despite the progress made, research on potential biomarkers in DFUs is still in its early stages. Continuous efforts in this field will not only yield new insights into DFU treatment but also enhance prevention and management strategies, ultimately improving the quality of care for patients with DFUs [125] (Table 2).

### 4.6. MS-Based Research in Diabetic Eye Disease

Diabetic retinopathy (DR) is a major microvascular complication of DM and the leading cause of vision loss in working-age adults worldwide. According to the International Diabetes Federation (2015), it is estimated that by 2030 the prevalence of DR and sight-threatening DR will increase to 191.0 million and 56.3 million, respectively. Furthermore, retinopathy can serve as a biomarker for vascular disease risk in asymptomatic diabetic individuals, indicating an elevated risk of life-threatening systemic vascular complications [126]. In a recent study [113], GC-MS was employed to analyze tear fluid samples from 41 T2DM patients with DR as well as 21 non-diabetic patients. The study revealed a significant prominence of D-glutamate and its metabolism in the proliferative diabetic retinopathy (PDR) group compared to the non-diabetic group. These metabolites identified in tear fluid have the potential to serve as potential biomarkers in the analysis of diabetic retinopathy. The validation of new metabolite biomarkers in the study involved data processing, OPLS-DA analysis, permutation testing, and pathway analysis. These steps ensured the accuracy and reliability of the identified metabolites and provided insights into their potential role in PDR. In a metabolomics study of plasma from patients with diabetic ophthalmopathy, Chen et al. [114] identified elevated levels of 2-deoxyribonucleic acid, 3,4-hydroxybutyric acid, erythritol, gluconic acid, and ribose using the GC-MS method. These elevated metabolites were validated in an independent sample set, indicating their potential as biomarkers for discriminating diabetic ophthalmopathy. Xuan et al. [115] conducted a study involving 461 participants, employing a combination of LC-MS and GC-MS techniques to analyze diabetes-related metabolic differential substances. They identified a total of 348 metabolites with intergroup differences, among which 12-hydroxy eicosatetraenoic acid and 2-piperidone were significantly elevated in diabetic ophthalmopathies. A binary logistic regression analysis resulted in an AUC area of 0.946 for the combination of these markers, with a diagnostic sensitivity and specificity of 0.894 and 0.919, outperforming the glycated hemoglobin marker (0.657, 0.686). Through a subsequent validation of the two markers in a separate cohort (444 participants), this approach ensured the reliability and reproducibility of the identified biomarkers for DR diagnosis. These results demonstrated the marker combination’s superior diagnostic power compared to the glycated hemoglobin index (0.392, 0.760). Comparisons between different stages of diabetic eye disease further confirmed the marker combination’s effectiveness, providing a promising new approach for diagnosing diabetic eye disease. (Refer to Table 2 for additional details.)

The studies analyzed different biosamples, such as tears, plasma, and serum, using advanced metabolomics techniques and employed methods like liquid chromatography–mass spectrometry and multiplatform metabolomics to identify and quantify specific metabolites associated with diabetic retinopathy. Following identification, statistical analysis was used to compare the levels of these metabolites between diabetic patients with and without retinopathy, ensuring the reliability of these biomarkers for distinguishing disease states. These rigorous validation methods enhance the credibility of the identified biomarkers in the clinical assessment of diabetic retinopathy.

## 5. Conclusions

This review describes how advanced MS technologies, including GC-MS/MS, LC-MS/MS, and UPLC-ESI-Q-TOF-MS/MS, have improved our understanding of diabetes at the molecular level. It details various studies that employed advanced MS to identify and quantify potential biomarkers associated with diabetes and its complications, demonstrating the versatility and precision of advanced MS in clinical diagnostics and metabolic research. The article highlights the application of MS in diabetes mellitus and its complications, such as gestational diabetes mellitus, diabetic peripheral neuropathy, diabetic retinopathy, diabetic nephropathy, diabetic encephalopathy, and diabetic cardiomyopathy. It also describes how MS-based metabolomics has enabled the identification of unique metabolic profiles and potential biomarkers, offering new insights into the disease’s pathogenesis and progression. For example, the results of the study showed that arginine was useful in the development of GDM to accurately differentiate between GDM and normoglycemia as well as in the early detection of GDM and that it also helps to predict increased risk of T2DM in women. The identification of markers such as 394 α-hydroxybutyric acid has been instrumental in pioneering early detection methodologies for individuals exhibiting insulin resistance and impaired glucose tolerance within non-diabetic cohorts. The discovery of syndrome-related biomarkers holds particular significance for personalized treatment approaches. Biomarkers associated with traditional Chinese medicine (TCM) syndromes can aid in clinical diagnosis, contribute to the modernization of TCM, and provide valuable insights into the pathogenesis of diabetes. However, we must accept that different metabolomic techniques, including LC-MS and GC-MS, use unique biomarker databases such that it is difficult to make direct comparisons, which makes cross-study comparisons challenging. Currently, there is no superior diagnostic or early warning marker for diabetes in clinical practice.

Mass spectrometry (MS) and tandem mass spectrometry (MS/MS) techniques offer significant advantages in metabolomics research, including (1) high sensitivity and specificity: MS and MS/MS technologies are capable of detecting metabolites at extremely low concentrations, which is crucial for identifying and quantifying rare or low-abundance metabolites within complex biological samples. Specificity is further enhanced by MS/MS through the selection and fragmentation of precursor ions, allowing for accurate identification of target molecules; (2) broad dynamic range: these technologies can detect metabolites across a wide concentration range, enabling the analysis of both high- and low-abundance metabolites, thus providing comprehensive metabolomic coverage; (3) structural elucidation capabilities: particularly with MS/MS, the fragmentation of molecules yields vital information regarding molecular structures. This is invaluable for the identification and structural characterization of unknown metabolites, facilitating the discovery of new metabolic pathways and biomarkers; (4) accurate quantitative analysis: MS techniques, especially when used in conjunction with internal standards, allow for precise quantification of metabolites. This is essential for understanding metabolic changes and disease mechanisms; (5) simplified sample preparation: compared to some traditional biochemical analysis methods, MS and MS/MS generally require fewer sample preparation steps and can directly analyze complex biological matrices, such as blood, urine, and tissue extracts; (6) high-throughput analysis capability: suitable for high-throughput analysis, MS technologies can process and analyze a large number of samples in a short period, which is crucial for large-scale metabolomics studies and biomarker screening; (7) versatility and flexibility: MS and MS/MS are applicable to a wide range of analytical targets, including small-molecule metabolites, proteins, and lipids, serving diverse research fields and objectives. Despite these advantages, MS and MS/MS also have limitations, such as high equipment costs, technical demands, and complex data analysis. Therefore, the choice of analytical methods should be determined based on specific research needs and resource availability.

The continuous evolution of advanced MS-based technologies for biomarker identification promises to enable more detailed and comprehensive metabolic profiling in the field of metabolomics. Future research directions include the integration of multi-omics approaches, where MS-based metabolomics is combined with genomics, proteomics, and transcriptomics to provide a holistic view of the biological processes underpinning diabetes mellitus and its complications. The potential of advanced MS-based metabolomics technologies in personalized medicine is particularly promising, as they allow the identification of individual-specific metabolic profiles, contributing to tailored therapeutic strategies that are more effective and have fewer side effects. Furthermore, advancements in MS technology coupled with artificial intelligence could lead to the development of predictive models for early diagnosis, risk assessment, and monitoring of disease progression in diabetes. These are integral to enhancing our understanding, diagnosis, and treatment of diabetes and its complications. The ongoing advancements in advanced MS-based biomarker identification for metabolomics technology and methodologies are set to further deepen our insights into diabetes mellitus and its complications, paving the way for innovative therapeutic strategies and improving the quality of life for individuals affected by diabetes.

## Figures and Tables

**Table 1:** Mass spectrometry ionization source types.

Ionization Source Type	Characteristics	Applicable Compounds	Instrument Type
**Electron Ionization (EI)**	Hard ionization, generates molecular ions	Suitable for thermally stable, volatile substances	Gas chromatography mass spectrometer (GC-MS)
**Chemical Ionization (CI)**	Soft ionization	Suitable for volatile, thermally stable substances	Gas chromatography mass spectrometer (GC-MS)
**Electrospray Ionization (ESI)**	Soft ionization source at atmospheric pressure	Suitable for less volatile, thermally unstable compounds	Liquid chromatography mass spectrometer (LC-MS) or capillary electrophoresis mass spectrometer (CE-MS)
**Atmospheric Pressure Chemical Ionization (APCI)**	Soft ionization; ionization of oxygen or nitrogen with a corona needle	Suitable for volatile, thermally stable substances	Liquid chromatography mass spectrometer (LC-MS)
**Matrix-Assisted Laser Desorption Ionization (MALDI)**	Soft ionization; the matrix cocrystallizes with the compound, and the compound ion is produced by laser hitting the matrix	Suitable for large molecules	Mass spectrometer (MS)
